# Adsorptive Features of Magnetic Activated Carbons Prepared by a One-Step Process towards Brilliant Blue Dye

**DOI:** 10.3390/molecules28041821

**Published:** 2023-02-15

**Authors:** Victoria X. Nascimento, Carlos Schnorr, Sabrina F. Lütke, Maria C. F. Da Silva, Fernando Machado Machado, Pascal S. Thue, Éder C. Lima, Julien Vieillard, Luis F. O. Silva, Guilherme L. Dotto

**Affiliations:** 1 Research Group on Adsorptive and Catalytic Process Engineering (ENGEPAC), Federal University of Santa Maria, Av. Roraima, 1000-7, Santa Maria 97105-900, RS, Brazil; 2 Department of Civil and Environmental, Universidad De La Costa, Calle 58 # 55-66, Barranquilla 080002, Atlántico, Colombia; 3 Technology Development Center, Federal University of Pelotas-UFPEL, Gomes Carneiro St., Pelotas 96010-610, RS, Brazil; 4 Environmental Science Graduate Program, Engineering Center, Federal University of Pelotas (UFPel), 989 Benjamin Constant St., Pelotas 96010-020, RS, Brazil; 5 Institute of Chemistry, Federal University of Rio Grande do Sul-UFRGS, Av. Bento Gonçalves 9500, P.O. Box 15003, Porto Alegre 91501-970, RS, Brazil; 6 Laboratoire de Chimie Organique, Normandie Université, UNIROUEN, INSA Rouen, CNRS, COBRA (UMR 6014), 27000 Evreux, France

**Keywords:** adsorption, brilliant blue, kinetic, sawdust, simulated effluent

## Abstract

Water pollution by dyes has been a major environmental problem to be tackled, and magnetic adsorbents appear as promising alternatives to solve it. Herein, magnetic activated carbons were prepared by the single−step method from Sapelli wood sawdust, properly characterized, and applied as adsorbents for brilliant blue dye removal. In particular, two magnetic activated carbons, MAC1105 and MAC111, were prepared using the proportion of biomass KOH of 1:1 and varying the proportion of NiCl_2_ of 0.5 and 1. The characterization results demonstrated that the different proportions of NiCl_2_ mainly influenced the textural characteristics of the adsorbents. An increase in the surface area from 260.0 to 331.5 m^2^ g^−1^ and in the total pore volume from 0.075 to 0.095 cm^3^ g^−1^ was observed with the weight ratio of NiCl_2_. Both adsorbents exhibit ferromagnetic properties and the presence of nanostructured Ni particles. The different properties of the materials influenced the adsorption kinetics and equilibrium of brilliant blue dye. MAC111 showed faster kinetics, reaching the equilibrium in around 10 min, while for MAC1105, it took 60 min for the equilibrium to be reached. In addition, based on the Sips isotherm, the maximum adsorption capacity was 98.12 mg g^−1^ for MAC111, while for MAC1105, it was 60.73 mg g^−1^. Furthermore, MAC111 presented the potential to be reused in more adsorption cycles than MAC1105, and the use of the adsorbents in the treatment of a simulated effluent exhibited high effectiveness, with removal efficiencies of up to 90%.

## 1. Introduction

Due to its pollution and scarcity, the water crisis has generated widespread concern worldwide. Several complex contaminants from industrial and domestic activities are indirectly or directly released into water bodies [1]. In this context, dyes are the main organic contaminants [2]. These compounds are widely used in textile, leather, cosmetics, plastic, pharmaceutical, food processing, and other industrial sectors [3]. Currently, more than 100,000 commercial dyes are available, and the estimated annual production of synthetic dyes is about 30,000 tons [2]. A large part of these dyes is usually discharged into water bodies through untreated wastewater, a considerable pollution source [4]. In general, dyes have a complex chemical construction, making their degradation difficult, and consequently, they remain longer in the environment [2]. The presence of dyes in water bodies can affect the photosynthetic activity of aquatic life [5,6]. In addition, it can affect some conditions, such as biological and chemical oxygen demands, dissolved oxygen concentration, pH, and the life of aquatic animals and their predators [2]. Thus, the treatment of wastewater containing dyes is a very relevant issue.

Several technologies have been used to remove dyes from wastewater, such as advanced oxidation processes [7], flocculation/coagulation [8], biological treatments [9], ion exchange [10], and adsorption [11]. Among these methods, adsorption is a promising technique for removing colored contaminants from wastewater [12,13]. Adsorption is promising since it has advantages such as applicability in different scenarios, ease of implementation, low space requirement, design simplicity, economic feasibility, and high efficiency [13,14].

In adsorption operations, activated carbon (AC) is widely used for removing inorganic and organic contaminants from aqueous solutions [15,16]. This adsorbent presents high surface area, pore volume, and abundant surface functional groups [17]. In parallel, the utilization of lignocellulosic wastes as precursor materials for AC production is a viable alternative due to its low cost, availability, and ecological suitability [18,19,20]. In previous literature, several studies have been carried out on developing AC from different lignocellulosic wastes such as pecan nutshells [21], tangerine seed [22], banana peel [23], apple peel [24], pistachio wood wastes [25], and teak wood sawdust [26]. Specifically, sawdust, a lignocellulosic waste obtained from various woodworking operations, is an accessible, abundant, and low-cost by-product and presents disposal problems [27,28]. Thus, the conversion of sawdust into adsorbents is a good alternative for managing this waste. In addition, the development of magnetic adsorbents has also been highlighted since it facilitates the separation of the solid from the liquid phase without requiring centrifugation [29,30,31,32]. Therefore, magnetic-activated carbons (MAC) have recently been proposed to remove contaminants [33,34,35,36,37]. Generally, these magnetic-activated carbons are prepared by co-precipitation, the activated carbon’s impregnation of the precursor material, followed by pyrolysis [37]. In the last procedure, carbonization, activation, and magnetization occur in a single step—an economic and convenient advantage [38]. However, regardless of the preparation method, evaluating different experimental conditions to prepare AC is fundamental to finding a material with relevant features for adsorption [12].

Herein, MACs were produced from Sapelli wood sawdust, characterized, and applied in dye adsorption from an aqueous solution. The precursor was first impregnated with NiCl_2_ and KOH; then, the impregnated materials underwent pyrolysis. The effect of different weight ratios of NiCl_2_ was investigated regarding their characteristics and performance on dye adsorption. Characterization was performed by scanning electron microscopy coupled with energy-dispersive X-ray spectroscopy (SEM/EDS), Fourier transform infrared spectroscopy (FTIR), N_2_ adsorption/desorption isotherms, X-ray diffraction (XRD), thermogravimetric analysis (TGA/DTG), and vibrating sample magnetometer (VSM). Adsorption studies involved kinetic and equilibrium experiments, regeneration/reuse tests, and tests with simulated industrial effluent.

## 2. Results and Discussion

### 2.1. Features of the Magnetic Activated Carbons

#### 2.1.1. Scanning Electron Microscopy Images

**Figure 1:**
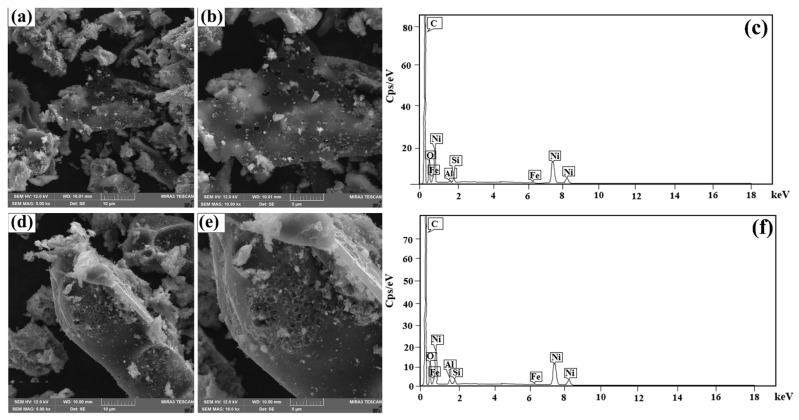
(**a**,**b**) SEM images and (**c**) EDS spectrum of MAC1105 and (**d**,**e**) SEM images and (**f**) EDS spectrum MAC111.

The SEM images of MAC1105 and MAC111, with magnifications of 5000× and 10,000×, are shown in Figure 1a,b and Figure 1d,e, respectively. It is possible to observe that both materials had an irregular surface, the presence of roughness and some grains, and cavities along their surfaces. In addition, MAC111 seems to have a higher quantity of cavities than MAC1105, which is the main difference observed between the surface morphology of the produced MACs. These cavities are favorable for application in adsorption processes because they enable the BB molecules to penetrate the adsorbent until reaching the pores [39,40]. Such cavities can be formed by the decomposition induced by KOH [41]. In addition, since both MACs were prepared with the same weight ratio of KOH, the higher quantity of cavities observed for MAC111 can be attributed to the higher weight ratio of NiCl_2_, which can cause the dehydration and decomposition of the lignocellulosic material [40]. This effect will be further explained in this paper.

Figure 1c shows the EDS spectrum of MAC1105, and Figure 1f shows the EDS spectrum of MAC111. For both MACs, carbon (C), oxygen (O), and nickel (Ni) were the major elements. The appearance of Si comes from the precursor material (Sapelli wood sawdust) used in the production of the adsorbents. Ni indicates that the Ni compounds formed during the pyrolysis were not eliminated with the acid wash but remained embedded in the carbon matrix. This behavior is important since these Ni compounds generate magnetization in the samples.

#### 2.1.2. Functional Groups on the Magnetic Activated Carbons Surface

**Figure 2:**
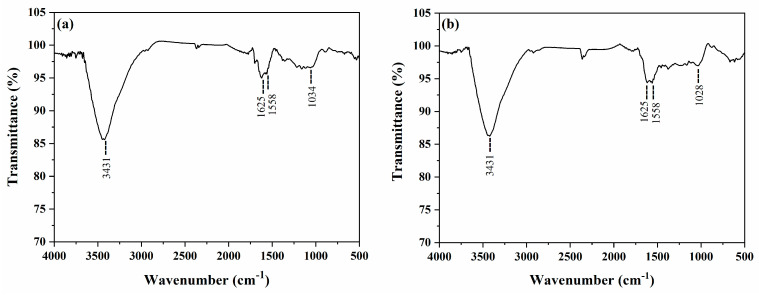
FTIR vibrational spectra of (**a**) MAC1105 and (**b**) MAC111.

Figure 2 shows the FTIR spectra of MAC1105 (Figure 2a) and MAC111 (Figure 2b). Bands at 3431, 1625, and 1558 cm^−1^ can be observed for both materials. The bands at 3431 cm^−1^ can be ascribed to the stretching vibrations of O–H bonds from alcohols, phenols, or carboxyls present as functional groups on the surface of the MACs and can also be ascribed to the presence of adsorbed water [42]. The bands at 1625 cm^−1^ can be attributed to the C=O bonds [43]. At 1558 cm^−1^, the bands observed can be attributed to the C=C stretching vibrations of the aromatic rings of the activated carbons’ structure [44]. Besides that, bands at 1034 e 1028 cm^−1^ can be observed for MAC1105 and MAC111, respectively, and are due to the C–O stretching vibrations of hydroxyl in alcohols, phenols, or carboxyls [45].

#### 2.1.3. Textural Characteristics

**Figure 3:**
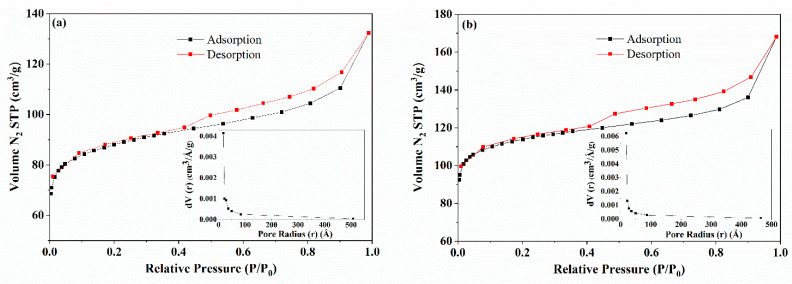
Nitrogen adsorption/desorption isotherms and the BJH pore size distribution of (**a**) MAC1105 and (**b**) MAC111.

Figure 3 shows the N_2_ adsorption/desorption isotherms and the BJH desorption pore size distributions for MAC1105 (Figure 3a) and MAC111 (Figure 3b). According to the IUPAC classification of adsorption isotherms, the adsorption isotherms of both materials were Type IV, accompanied by hysteresis [46]. Type IV isotherm is a characteristic of mesoporous materials. The adsorption hysteresis exhibited by both MACs was Type H4. This type of hysteresis loop is characteristic of micro/mesoporous carbonaceous materials [46]. From the pore size distribution, mesopores are observed for both materials.

Table 1 presents both materials’ surface area, total pore volume, and average pore size. It can be observed that MAC111 presented a higher BET surface area and higher total pore volume than MAC1105. This behavior is because the salt KOH used as the activating agent can react with the carbonaceous material. Since both MAC1105 and MAC111 were prepared with the same weight ratio of KOH, the superior features presented by MAC111 may be due to the higher weight ratio of NiCl_2_ used to prepare this material. Therefore, in addition to generating magnetization, NiCl_2_ can affect the textural characteristics of the samples. Similar behavior was observed by Thue et al. [37]. The authors prepared magnetic activated carbons from tucumã seed using ZnCl_2_ and NiCl_2_. They found out that the increase in the weight ratio of NiCl_2_ led to an increase in the surface area and total pore volume of the obtained material. In addition, several other authors found that increasing the proportion of transition metal chlorides, such as ZnCl_2_, FeCl_3_, CoCl_2_, and CuCl_2_, led to improved textural properties [40,46,47,48,49,50]. The transition metals impregnated in the precursor material are cross-linked to the surface functional groups of the biomass, promoting the chemical dehydration and decomposition of the lignocellulosic material during pyrolysis. After acid washes, the transition metals are removed, and the previously occupied spaces become free, forming the pores. Therefore, with higher weight rations of these salts, more transition metal ions are available, improving the formation of the pore structure [40,48,49,50]. In the present work, although the presence of Ni in the carbon matrix was shown by the EDS spectra (Figure 1), part of it may have been eliminated with the acid wash. Therefore, with a higher weight ratio of NiCl_2,_ more pores may have been created and, subsequently, become free with the acid wash. This behavior may explain the higher surface area and total pore volume observed for MAC111.

Regarding the average pore sizes, values of 3.69 and 3.60 nm were found for MAC1105 and MAC111, respectively (Table 1). According to IUPAC, the pores of an adsorbent can be classified as micropores (inner diameter < 2 nm), mesopores (2 nm ≤ inner diameter ≤ 50 nm), and macropores (inner diameter > 50 nm) [46]. Therefore, both MACs can be classified as mesoporous materials, corroborating the isotherms in Figure 3.

#### 2.1.4. Thermal Analysis

**Figure 4:**
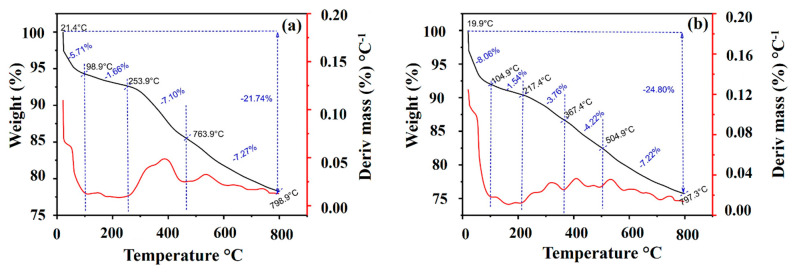
TGA and DTG curves of (**a**) MAC1105 and (**b**) MAC111.

Figure 4 shows the thermal profile of the produced MACs. The thermal behavior was evaluated from room temperature to 800 °C under an N_2_ atmosphere. The curve obtained for MAC1105 can be divided into four regions, while the curve obtained for MAC111 can be divided into five regions. The first region of weight loss was 21.4–98.9 °C and 19.9–104.9 °C for MAC1105 and MAC111, respectively, and can be attributed to the evaporation of moisture [37,51]. The temperature range for the second region of weight loss was 98.9–253.9 °C and 104.9–217.4 °C for MAC1105 and MAC111, respectively. This stage corresponds to the loss of water from the channels and pores of the material [37,51]. The weight loss sum until these temperatures were 7.37% for MAC1105 and 9.60% for MAC111. The third region of weight loss varied from 253.9–463.9 °C MAC1105. For MAC111, a third stage of weight loss occurred from 217.4–367.4 °C, and a fourth stage occurred from 367.4–504.9 °C. These stages of weight loss can be assigned to the decomposition of oxygenated functional groups on the activated carbon surface [39]. The last stage for MAC1105 (fourth stage) was 763.9–798.9 °C, and the last stage for MAC111 (fifth stage) was 504.9–793.3 °C. These weight-loss stages correspond to the carbon matrix’s oxidation [51]. The total weight loss was 21.74% for MAC1105 and 24.80% for MAC111.

#### 2.1.5. X-ray Diffraction

**Figure 5:**
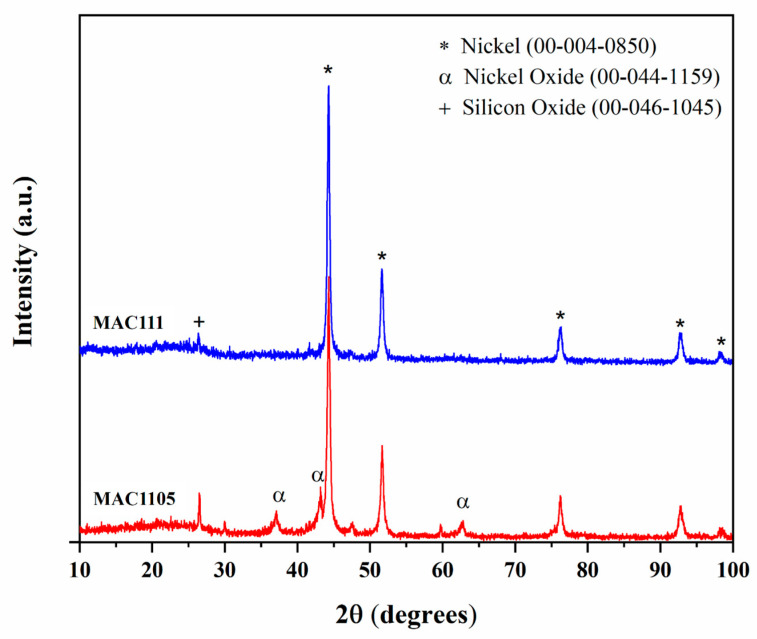
XRD patterns of MAC1105 and MAC111.

Figure 5 shows the XRD diffractograms of MAC1105 and MAC111. Both samples show patterns of metallic Ni (crystalline system: cubic; JCPDS Card 00-004-0850). According to Thue et al. [37], the formation of metallic Ni occurs due to the reduction of the previously impregnated Ni^2+^ to Ni^0^. During pyrolysis, this reaction occurs at high temperatures and in the presence of reducing gases, such as H_2_ and CH_4_. MAC1105 also shows patterns of nickel oxide (NiO; crystalline system: rhombohedral; JCPDS Card 00-044-4459). These results also confirm that the Ni was not completely eliminated during the acid wash. Besides that, both MACs show patterns of silicon oxide (SiO_2_; crystalline system: hexagonal; JCPDS Card 00-046-1045). The presence of SiO_2_ in the samples comes from the Sapelli wood sawdust.

Nanostructured Ni particles with average crystallite sizes of 18.96 and 25.34 nm for MAC1105 and MAC111, respectively, were obtained (calculated using Scherrer’s equation).

#### 2.1.6. Magnetic Features

**Figure 6:**
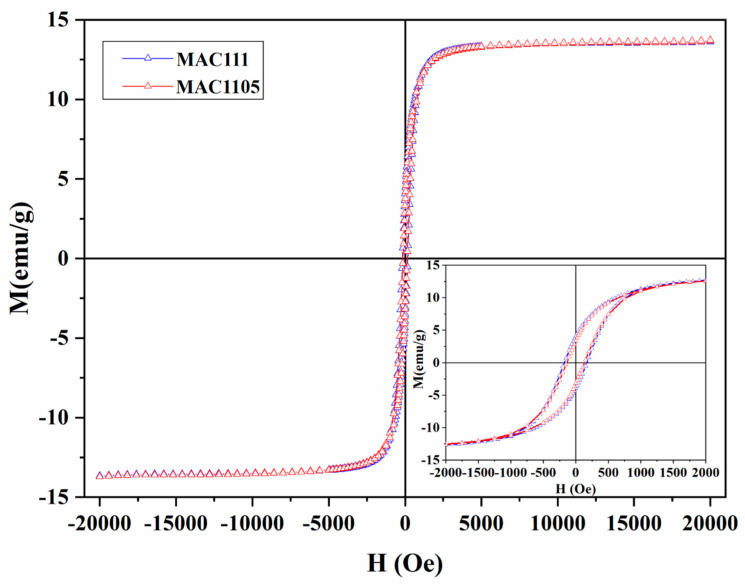
M-H hysteresis loops of MAC1105 and MAC111 at room temperature. The inset is a magnified view of the M-H curves.

Magnetization curves of the MACs are shown in Figure 6, and the values of the hysteresis parameters are shown in Table 2. As can be seen, both exhibit ferromagnetic properties, with coercivity (H_C_) values of 150.2 Oe and 200.2 Oe and remanence (M_R_) values of 3.2 emu g^−1^ and 4.1 emu g^−1^ for MAC1105 and MAC111, respectively. These are interesting values for this kind of material [37,52,53]. On the other hand, the saturation magnetization (M_S_) value, 13.6 emu g^−1^ for both MACs, is slightly lower than that which the literature reported [37,52,53]. Despite the relatively low M_S_ value, the MACs produced can be easily moved and controlled in aqueous media by external magnetic fields.

### 2.2. Adsorption Results

#### 2.2.1. Kinetic Profiles of BB Adsorption

**Figure 7:**
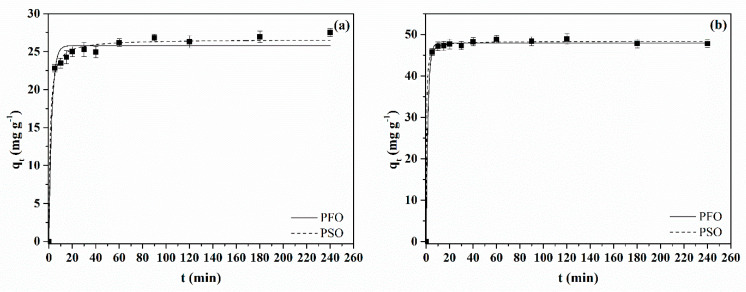
The kinetic curve of BB dye adsorption onto (**a**) MAC1105 and (**b**) MAC111.

The kinetic behavior of BB dye adsorption was studied for both MACs with an initial adsorbate concentration of 50 mg L^−1^ and pH 4. The adsorption capacity curves as a time function are shown in Figure 7a,b for MAC1105 and MAC111, respectively. It is possible to observe that the curve obtained for MAC111 was characterized by a faster adsorption rate. MAC111 reached equilibrium in around 10 min. For MAC1105, the equilibrium was reached in around 60 min. This behavior can be explained due to the higher total pore volume presented by MAC111 (Table 1). A high pore volume can enhance the kinetic of adsorption by allowing a faster diffusion rate of the dye molecules inside the pores to the adsorption sites [54].

The experimental kinetic data were fitted to the pseudo-first-order (PFO) and pseudo-second-order (PSO) models. The parameters of the models are depicted in Table 3. Based on the higher values of *R*^2^ and *R*^2^*_adj_* and the lower values of *ARE*, it can be concluded that the PSO model was the more suitable to represent the BB dye adsorption kinetic for both MACs. Furthermore, Table 3 confirms the faster kinetic and the higher adsorption capacity of MAC111 because *k*_2_ and *q*_2_ for this adsorbent were around two times higher concerning the same parameters for MAC1105. For comparison, several authors demonstrated that the PSO model was more suitable for representing the kinetic data of anionic dye adsorption [21,55,56,57].

#### 2.2.2. Adsorption Isotherms

**Figure 8:**
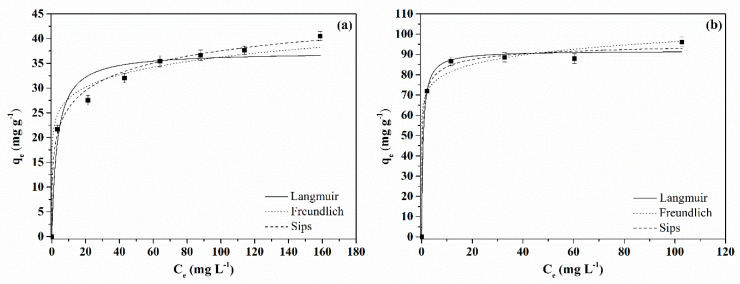
Equilibrium isotherms of BB blue dye adsorption onto (**a**) MAC1105 and (**b**) MAC111.

The equilibrium study for BB dye adsorption was carried out at 25 °C with initial adsorbate concentrations from 0 to 200 mg L^−1^ and pH 4. Figure 8 shows the equilibrium curves obtained for MAC1105 (Figure 8a) and for MAC111 (Figure 8b). According to Giles classification [58], the isotherms obtained for MAC1105 and MAC111 are typical type L1 and L2 isotherms, respectively. L-type isotherms indicate a high affinity between the adsorption sites of the adsorbent and the adsorbate molecules. Besides that, it is possible to observe (Figure 8b) that MAC111 presented a higher adsorption capacity than MAC1105. This result can also be explained due to the superior textural features exhibited by MAC111 (Table 1). The higher surface area presented by MAC111 makes more surface active sites available to capture the adsorbate molecules.

Langmuir, Freundlich, and Sips models were used to interpret the equilibrium curves. Table 4 shows the equilibrium parameters. Based on the *R*^2^, *R*^2^*_adj_*, and *ARE* values, it is possible to observe that only the Sips model adequately represents the equilibrium data for MAC1105. On the other hand, for MAC111, all three models presented a good fit. However, the Sips model presented slightly higher *R*^2^ and *R*^2^*_adj_* values and a slightly lower *ARE* value. Therefore, the experimental equilibrium data for both MACs established that the Sips model was the best choice.

For comparison, Table 5 shows the maximum adsorption capacity of different adsorbents for BB dye adsorption reported in the literature. It can be seen that the values found in the present study are quite promising, and further studies on the adsorption conditions are required to increase the adsorption capacity.

#### 2.2.3. Regeneration and Reuse Study

**Figure 9:**
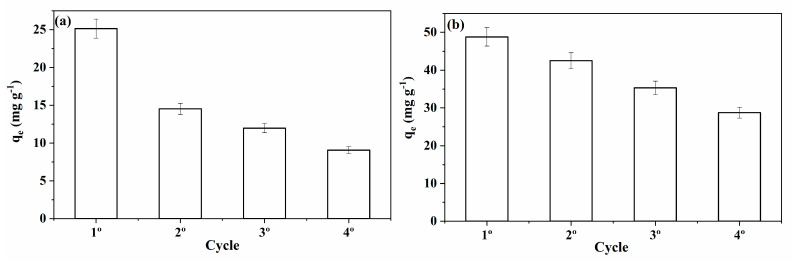
Reuse cycles of (**a**) MAC1105 and (**b**) MAC111.

Different chemical agents in different concentrations were tested for regenerating the MACs. Table 6 shows the equilibrium adsorption capacity for the second cycle of adsorption. It can be seen that the best results were obtained using NH_4_OH in the concentration of 0.5 mol L^−^^1^ for both MACs. Therefore, additional adsorption/regeneration cycles were performed using this regeneration condition to access the potential of reusing both materials. Figure 9 shows the equilibrium adsorption capacity of BB dye for four cycles using MAC1105 (Figure 9a) and MAC111 (Figure 9b). It is possible to notice that the adsorption capacity gradually decreased over the cycles. This trend suggests that some BB dye molecules were not removed from the adsorbent surface upon regeneration. Therefore, the active sites occupied by these molecules become unavailable for adsorption in the next cycle.

It also can be observed that MAC111 showed a smaller decrease in adsorption capacity over the cycles than MAC1105 (Figure 9). In addition, the adsorption capacity observed for MAC111 at the end of the fifth cycle (around 29 mg g^−^^1^) is higher than the adsorption capacity observed for MAC1105 even in the first cycle (around 25 mg g^−^^1^). Overall, the results showed that MAC111 has a higher potential to be reused in more adsorption cycles, directly affecting the operating costs.

#### 2.2.4. Application of MACs to Treat a Simulated Effluent

**Figure 10:**
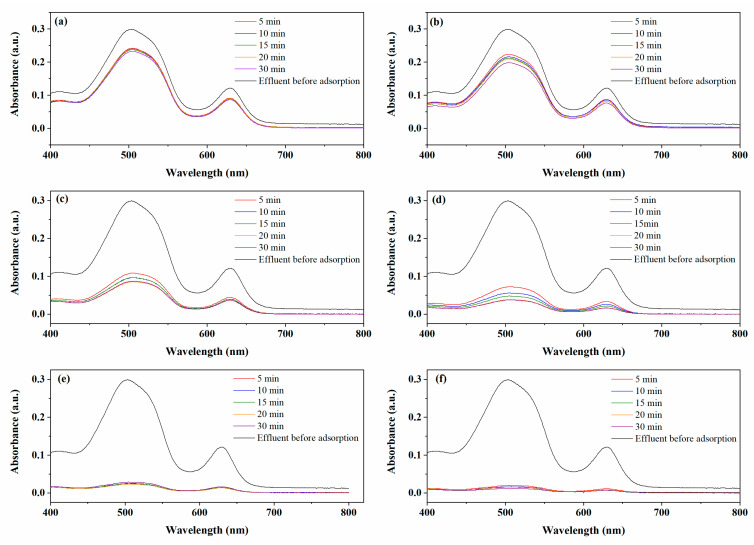
Absorption spectra of simulated effluent before and after adsorption with 1 g L^−^^1^: (**a**) MAC1105 and (**b**) MAC111; 5 g L^−^^1^: (**c**) MAC1105 and (**d**) MAC111; 10 g L^−^^1^: (**e**) MAC1105 and (**f**) MAC111.

A simulated effluent was prepared from a grape drink mix to verify the applicability of the MACs for removing BB dye in a complex matrix. The MACs were evaluated regarding the removal efficiency using different adsorbent dosages (1, 5, and 10 g L^−^^1^) and contact times (5–30 min). The visible spectra (400–800 nm) of the simulated effluent before and after the adsorption tests are shown in Figure 10. It was observed that the adsorption was fast since the removal efficiencies were virtually the same for all contact times studied. However, the removal efficiency obtained using a dosage of 1 g L^−^^1^ was below 40%. The active sites are easily saturated with low adsorbent dosages since the simulated effluent contains several compounds other than the BB dye. However, using higher adsorbent dosages, high removal efficiencies were obtained. In 30 min, MAC1105 and MAC111 were capable of removing, respectively, 73% and 88% using the adsorbent dosage of 5 g L^−^^1^, and 93% and 95% using the adsorbent dosage of 10 g L^−^^1^. These results indicated that the MACs are promising adsorbents for treating effluents from the food industry containing BB dye.

## 3. Material and Methods

### 3.1. Materials

Sapelli wood sawdust (*E. cylindricum*), used as a precursor material for producing the magnetic activated carbons, was obtained from sawmills in Ngaoundere (Cameroon). The sawdust was milled, obtaining particle sizes lower than 250 μm. The anionic dye Brilliant Blue (BB, CI 42090, molar weight 792.8 g mol^−1^, *λ_max_* 630.0 nm) was supplied by Duas Rodas company (Brazil). Figure S1 (Supplementary Material) shows the chemical structure of the BB dye obtained by the Chemsketch software. All other reagents used were of analytical grade.

### 3.2. Preparation of the Magnetic Activated Carbons

In the MACs preparation, NiCl_2_ produced magnetic features, and KOH was used as an activating agent to increase pore size and specific surface area. Initially, 100.0 g of the Sapelli wood sawdust was mixed with 100.0 g of KOH and 50.0 or 100.0 g of NiCl_2_ (weight ratios of 1:1:0.5 and 1:1:1, respectively). Then, about 50 mL of distilled water was added to the mixtures, which were stirred with a magnetic stirrer at 90 °C for 2 h. The homogeneous pastes formed were oven-dried at 105 °C for 8 h, and after drying, each paste was introduced into a quartz reactor in a conventional furnace (Sanchis, Brazil). The heating was carried out from room temperature to 600 °C at a heating rate of 10 °C min^−1^ and an inert gas (N_2_) flow rate of 150 mL min^−1^. After reaching the final temperature, it was maintained for 30 min. Then, the furnace was turned off and kept under the inert gas flow until it reached a temperature below 200 °C. After cooling, the obtained materials were washed with a 0.1 mol L^−1^ HCl solution under a reflux system at around 80 °C for 2 h. Subsequently, the materials were exhaustively washed with distilled water until the pH of the washing waters attained the pH of distilled water (pH 6–7). Finally, the materials were oven-dried at 105 °C for 8 h. The materials obtained were MAC1105 and MAC111, according to the weight ratios of Sapelli sawdust: KOH: NiCl_2_ of 1:1:0.5 and 1:1:1, respectively.

### 3.3. MACs Characterization

The textural properties (BET surface area, total pore volume, and average pore size) were obtained from N_2_ adsorption/desorption isotherms at 77 K in a volumetric adsorption analyzer (Micromeritcs, ASAP 2020, USA) using BET and BJH methods.

The surface morphologies of the materials were obtained by scanning electron microscopy (SEM) coupled with energy-dispersive X-ray spectroscopy (EDS) (Tescan, MIRA 3, Czech Republic). The working voltage was 12 kV, and the magnifications were 5000× and 10,000×.

Investigations concerning the surface functional groups were realized by Fourier transform infrared spectroscopy (FTIR) (Shimadzu, Prestige 21210045, Japan). The spectra were obtained with a resolution of 4 cm^−1^ in the range from 4000 to 400 cm^−1^ by diffuse reflectance technique with KBr.

Thermogravimetric (TGA) and derivative thermogravimetric (DTG) curves of the magnetic activated carbons were obtained in a TA instrument (Netzsch, STA 449 F3 Jupiter^®^, Germany). The analysis was carried out in the following conditions: temperature from 20 °C to 800 °C, a heating rate of 25 °C min^−1^, and an N_2_ flow rate of 50 mL min^−1^.

The crystalline nature of the MACs was accessed by Powder X-Ray Diffraction (XRD) (Rigaku, Miniflex 300, Japan). The instrument was operated at 30 kV and 10 mA with Cu Kα radiation (*λ* = 1.541861 Å). Measurements were done over the 10° ≤ 2θ ≤ 100°, using a scanning step of 0.06° s^−1^. Scherrer’s equation obtained the Ni particles’ average crystallite size (D) in the magnetic activated carbons.

The magnetic properties were investigated at room temperature utilizing a vibrating-sample magnetometer (VSM) (MicroSense, EZ9, USA), performing from −20 kOe to +20 kOe.

### 3.4. Kinetic and Equilibrium Adsorption Experiments

Adsorption studies of BB dye from aqueous solutions were performed to evaluate the adsorption performance of the MACs. BB dye is extensively used in the food industry and can be found in various foods such as drink mixes, ice creams, candies, and gelatins.

For the adsorption assays, 20 mL of the BB dye solution at pH 4.0 (adjusted with 0.1 mol L^−1^ HCl solution) were added in Erlenmeyer flasks with 0.02 g of the MAC (adsorbent dosage of 1 g L^−1^). The flasks were stirred in a thermostatic agitator (Solab, SL222, Brazil) at 25 °C and 150 rpm. The kinetic study was performed with an initial BB dye concentration of 50 mg L^−1^ at set intervals (0–240 min). The equilibrium study was carried out with initial BB dye concentrations from 0 to 200 mg L^−1^ until it reached equilibrium. Afterward, the adsorbent was separated from the liquid phase using a magnet. The remaining BB concentration in the liquid was measured by spectrophotometry (Biospectro SP-22, Brazil) at 630 nm. The adsorption results were represented according to the standard of our group (supplementary material S1).

### 3.5. Kinetic and Equilibrium Models

See Supplementary Materials Sections S2 and S3.

### 3.6. Regeneration and Reuse Experiments

Regeneration and reuse tests were conducted to verify the possibility of reusing the MACs. First, the MACs were loaded with BB dye at the following experimental conditions: initial dye concentration of 50 mg L^−1^, pH 4.0, an adsorbent dosage of 1 g L^−1^, 25 °C, stirring rate of 150 rpm, and 2 h. Then, the adsorbent was separated from the medium by a magnet and oven-dried at 105 °C for 8 h.

In the regeneration step, four different regeneration agents—namely sodium chloride (NaCl), sodium hydroxide (NaOH), ammonium hydroxide (NH_4_OH), and acetone (C_3_H_6_O)—in the concentrations of 0.5 and 1.0 mol L^−1^ were tested to desorb the BB dye from the adsorbent. In this step, 20 mL of the regeneration agent was added to Erlenmeyer flasks with the magnetic-activated carbon previously loaded with BB dye. The flasks were stirred for 2 h at 25 °C and a stirring rate of 150 rpm. Subsequently, the regenerated adsorbent was separated from the medium using a magnet and oven-dried at 105 °C for 8 h. After that, the regenerated magnetic activated carbons were used again for BB dye adsorption. The adsorption-regeneration cycle was realized several times using the regeneration agent that showed the best results.

### 3.7. Application in a Simulated Effluent

The simulated effluent was prepared using a grape drink mix from the local industry and presented a BB dye concentration of 52 mg L^−1^ and a pH of 3.0. The matrix of the simulated effluent was composed of sugars, dehydrated grape juice, vitamin C, acidulant (citric acid), acidity regulator (trisodium citrate), flavorings, sweeteners (potassium aspartame and acesulfame), anti-humectant (tricalcium phosphate), thickeners (sodium carboxymethylcellulose and xanthan gum) and dyes (brilliant blue, titanium dioxide derivatives, red 40, and indigotin blue). The experiments were carried out with 20 mL of the simulated effluent, a temperature of 25 °C, and a stirring rate of 150 rpm. Different adsorbent dosages (1, 5, and 10 g L^−1^) and contact times (5–30 min) were tested. Before and after the adsorption tests, the spectra were obtained in a UV-vis spectrometer (Shimadzu, UV240, Japan). The spectra were recorded from 400 to 800 nm, and the areas under the absorption bands were used to obtain the removal efficiencies.

## 4. Conclusions

In this work, magnetic activated carbons from Sapelli wood sawdust were successfully prepared, characterized, and applied in the adsorption of BB dye from an aqueous solution. The different weight ratios of NiCl_2_ mainly influenced the textural properties of the materials. The increase in the weight ratio of NiCl_2_ led to an increase in the surface area from 260.0 m^2^ g^−^^1^ (MAC1105) to 331.5 m^2^ g^−^^1^ (MAC111) and in the total pore volume from 0.075 cm^3^ g^−^^1^ (MAC1105) to 0.095 cm^3^ g^−^^1^ (MAC111). The average pore size remained virtually the same, around 3.6 nm, regardless of the weight ratio of NiCl_2_. Both MACs exhibit ferromagnetic properties at room temperature. Nanostructured Ni particles with average crystallite sizes of 18.96 nm (MAC1105) and 25.34 nm (MAC111) were observed. According to the FTIR spectra, very similar surface chemistry was obtained for both MACs exhibiting hydroxyl and carboxyl groups. Furthermore, a very similar thermal behavior was also observed for both materials.

From the adsorption study, important differences between the MACs were observed. MAC111 exhibited faster kinetics and reached equilibrium in around 10 min. For MAC1105, on the other hand, the equilibrium was reached only in 60 min. For both adsorbents, the pseudo-second-order model represented the kinetic data well. Regarding the adsorption isotherms, the Sips model satisfactorily represented the data. In addition, MAC111 showed a higher maximum adsorption capacity (*q_s_* = 98.12 mg g^−^^1^) than MAC1105 (*q_s_* = 60.73 mg g^−^^1^). The regeneration and reuse study showed that MAC111 had a higher potential to be reused in more adsorption cycles. Finally, MAC1105 and MAC111 were promising for treating a simulated effluent containing the BB dye, achieving removal efficiencies of 93% and 95%, respectively. These results demonstrated that the MACs were promising adsorbents for BB dye removal from an aqueous solution with good efficiency and easy magnetic separation. Besides that, it is noteworthy that MAC111 showed the best results due to its best features arising from the higher weight ratio of NiCl_2_ in its production. Nevertheless, further and more complete studies are required for application purposes, such as the effect of the adsorbent dosage, pH and temperature, and the adsorption thermodynamics.

## Figures and Tables

**Table 1:** Textural characteristics of the magnetic activated carbons.

Activated Carbon	BET Surface Area (m^2^ g^−1^)	Total Pore Volume (cm^3^ g^−1^)	Average Pore Size (nm)
MAC1105	260.0	0.075	3.69
MAC111	331.5	0.095	3.60

**Table 2:** Magnetic properties of the magnetic activated carbons.

Sample	Coercivity (H_C_, Oe)	Saturation Magnetization (M_S_, emu g^−1^)	Remanence (M_R_, emu g^−1^)
MAC1105	150.2	13.6	3.2
MAC111	200.2	13.6	4.1

**Table 3:** Kinetic parameters for the adsorption of BB dye.

Model	Activated Carbon	
	MAC1105	MAC111
PFO		
*q_1_* (mg g^−1^)	25.79	47.93
*k_1_* (min^−1^)	0.396	0.612
*R* ^2^	0.9785	0.9983
*R* ^2^ *_adj_*	0.9480	0.9959
*ARE* (%)	3.83	1.00
PSO		
*q*_2_ (mg g^−1^)	26.62	48.36
*k*_2_ (g mg^−1^ min^−1^)	0.035	0.072
*R* ^2^	0.9931	0.9991
*R* ^2^ *_adj_*	0.9832	0.9977
*ARE* (%)	2.12	0.70

**Table 4:** Equilibrium parameters for the adsorption of BB dye.

Model	Activated Carbon	
	MAC1105	MAC111
Langmuir		
*q_m_* (mg g^−1^)	37.32	91.80
*K_L_* (L mg^−1^)	0.314	1.655
*R* ^2^	0.9542	0.9946
*R* ^2^ *_adj_*	0.9359	0.9910
*ARE* (%)	7.58	2.28
Freundlich		
*K_F_* ((mg g^−1^)(mg L^−1^)^−1/nF^)	21.46	67.52
1/*nF*	0.114	0.077
*R* ^2^	0.9785	0.9927
*R* ^2^ *_adj_*	0.9699	0.9878
*ARE* (%)	5.79	2.44
Sips		
*q_S_* (mg g^−1^)	60.73	98.12
*K_S_* (L mg^−1^)	0.346	1.938
*m_S_*	0.335	0.483
*R* ^2^	0.9937	0.9957
*R* ^2^ *_adj_*	0.9890	0.9894
*ARE* (%)	2.94	2.24

**Table 5:** Adsorption capacities of different adsorbents for BB dye adsorption.

Adsorbent	Dosage (g L^−1^)	pH	T (°C)	*Adsorption capacity* (mg g^−1^)	Reference
MAC1105	1.0	4.0	25	60.7	This study
MAC111	1.0	4.0	25	98.1	This study
Chitosan vermiculite beads	5.0 ^a^	10.2	25	181.6	[59]
Hen feather	0.4 ^a^	2.0	30	317.0 ^b^	[60]
Magnetic tungsten disulfide/carbon nanotubes nanocomposite	0.3 ^a^	3.0	25	166.7	[61]
Bottom ash	4.0 ^a^	3.0	50	6.9 ^b^	[62]
De-oiled soya	2.0 ^a^	3.0	50	18.2 ^b^	[62]
Unmodified clay	10.0 ^a^	5.4	30	6.2	[63]
Iron-modified clay	10.0 ^a^	5.4	30	14.2	[63]

^a^ Calculated; ^b^ Original values converted to a mass base using the BB molar weight of 792.8 g mol^−^^1^.

**Table 6:** Regeneration test using different regeneration agents in different concentrations.

Activated Carbon	Regenerating Agent	Concentration of the Regenerating Agent (mol L^−1^)	q_e_ in the Second Cycle (mg g^−1^) ^a^
MAC1105	NaCl	0.5	6.05 ± 0.65
		1.0	5.80 ± 0.58
	NaOH	10.5	7.09 ± 0.74
		1.0	5.89 ± 0.63
	NH_4_OH	0.5	10.78 ± 1.01
		1.0	5.72 ± 0.69
	C_3_H_6_O	0.5	8.58 ± 0.95
		1.0	7.35 ± 0.71
MAC111	NaCl	0.5	34.96 ± 0.87
		1.0	30.59 ± 1.23
	NaOH	10.5	29.21 ± 0.91
		1.0	22.52 ± 1.13
	NH_4_OH	0.5	36.64 ± 0.87
		1.0	31.04 ± 0.92
	C_3_H_6_O	0.5	36.31 ± 1.04
		1.0	30.91 ± 0.98
	NaCl	0.5	34.96 ± 0.87

^a^ Mean ± standard deviation (n = 3).

## Data Availability

The data will be available on request.

## Supplementary Materials

The following supporting information can be downloaded at: https://www.mdpi.com/article/10.3390/molecules28041821/s1, Figure S1: Chemical structure of the brilliant blue dye; Section S1: Adsorption quantification; Section S2: Kinetic models; Section S3: Isotherm models [64,65,66,67].
