# Supplementary material for: Adsorptive Features of Magnetic Activated Carbons Prepared by a One-Step Process towards Brilliant Blue Dye
Source: Molecules. 2023 Feb 15;28(4):1821. doi: 10.3390/molecules28041821 (PMC9965938; doi:10.3390/molecules28041821)
Supplement: Supplementary file 1 [file molecules-28-01821-s001.zip › molecules-2194580-supplementary.pdf]

Supplementary Material

Adsorptive Features of Magnetic Activated Carbons Prepared by a One-Step Process towards Brilliant Blue Dye

Victoria X. Nascimento [1], Carlos Schnorr [2], Sabrina F. Lütke [1], Maria C. F. Da Silva [1], Fernando Machado Machado [3], Pascal S. Thue [4], Éder C. Lima [5], Julien Vieillard [6], Luis F. O. Silva [2,*] and Guilherme L. Dotto [1,6,*]

[1] Research Group on Adsorptive and Catalytic Process Engineering (ENGEPAC), Federal University of Santa Maria, Av. Roraima, 1000–7, Santa Maria 97105–900, RS, Brazil
[2] Universidad De La Costa, Calle 58 # 55–66, Barranquilla 080002, Atlántico, Colombia
[3] Technology Development Center, Federal University of Pelotas–UFPEL, Gomes Carneiro St., Pelotas 96010–610, RS, Brazil
[4] Environmental Science Graduate Program, Engineering Center, Federal University of Pelotas (UFPel), 989 Benjamin Constant St., Pelotas 96010–020, RS, Brazil
[5] Institute of Chemistry, Federal University of Rio Grande do Sul–UFRGS, Av. Bento Gonçalves 9500, P.O. Box 15003, Porto Alegre 91501–970, RS, Brazil
[6] Normandie Université, UNIROUEN, INSA Rouen, CNRS, COBRA (UMR 6014), 27000 Evreux, France
* Correspondence: lsilva8@cuc.edu.co (L.F.O.S.); guilherme.dotto@ufsm.br (G.L.D.)

Citation: Nascimento, V.X.; Schnorr, C.; Lütke, S.F.; da Silva, M.C.F.; Machado, F.M.; Thue, P.S.; Lima, É.C.; Vieillard, J.; Silva, L.F.O.; Dotto, G.L. Adsorptive Features of Magnetic Activated Carbons Prepared by a One-Step Process towards Brilliant Blue Dye. *Molecules* **2023**, *28*, x. https://doi.org/10.3390/xxxxx

Academic Editors: Jinpeng Li, Jun Xu, Bin Wang and Wenhua Gao

Received: 16 January 2023
Revised: 8 February 2023
Accepted: 11 February 2023
Published: 15 February 2023

Copyright: © 2023 by the authors. Licensee MDPI, Basel, Switzerland. This article is an open access article distributed under the terms and conditions of the Creative Commons Attribution (CC BY) license (https://creativecommons.org/licenses/by/4.0/).

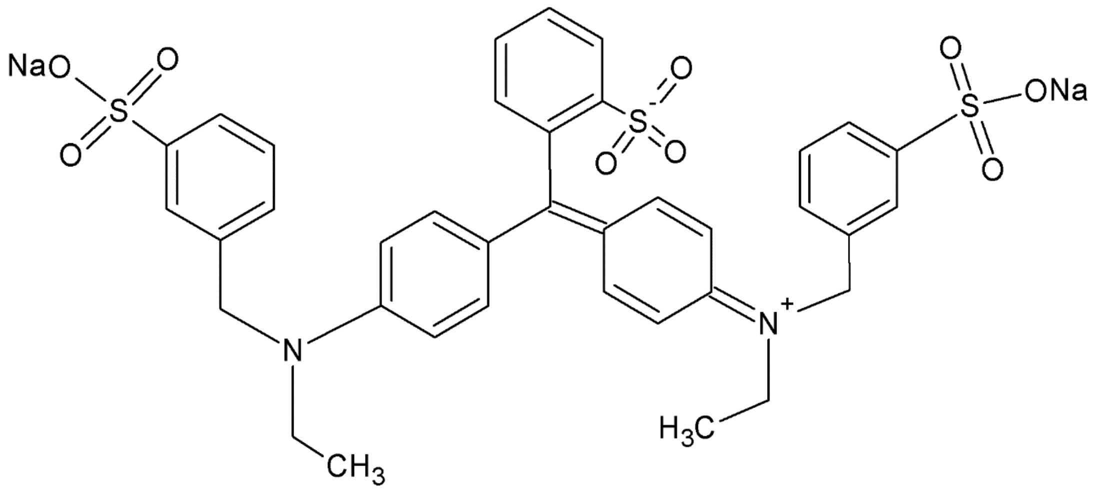

Figure S1. Chemical structure of the brilliant blue dye.

S1. Adsorption quantification

All assays were performed in replicate (n = 3) and blank tests were also carried out. The adsorption capacity at time t (q_t) and the equilibrium adsorption capacity (q_e) were determined by Eq. (1) and (2), respectively:

$$q_t = \frac{V(C_0 - C_t)}{m} \tag{1}$$

$$q_e = \frac{V(C_0 - C_e)}{m} \tag{2}$$

Where C_0 is the initial BB dye concentration (mg L^{-1}), C_t is the BB dye concentration in the liquid phase at time t (mg L^{-1}), C_e is the equilibrium BB dye concentration in the liquid phase (mg L^{-1}), m is adsorbent mass (g), and V is the volume of the solution (L).

S2. Kinetic models

The adsorption kinetic behavior onto the MACs was evaluated by the pseudo-first-order (PFO) and pseudo-second-order (PSO) models [1]. The mathematical equations of these models are shown in Eq. (3) and (4), respectively:

$$q_t = q_1(1 - exp(-k_1 t)) \tag{3}$$

$$q_t = \frac{t}{(1/k_2 q_2^{\,2}) + (t/q_2)} \tag{4}$$

Where q_t is the adsorption capacity at time t (mg g^{-1}), q_1 is the adsorption capacity predicted by the pseudo-first-order model (mg g^{-1}), q_2 is the adsorption capacity predicted by the pseudo-second-order model (mg g^{-1}), k_1 is the pseudo-first-order rate constant (min^{-1}), and k_2 is the pseudo-second-order rate constant (g mg^{-1} min^{-1}).

S3. Isotherm models

The adsorption equilibrium data were fitted to the Langmuir [2], Freundlich [3], and Sips [4] models, which are presented in the Eq. (5), (6), and (7), respectively:

$$q_e = \frac{q_m K_L C_e}{1 + K_L C_e} \tag{5}$$

$$q_e = K_F C_e^{1/nF} \tag{6}$$

$$q_e = q_S \frac{K_S C_e^{\,m_S}}{1 + K_S C_e^{\,m_S}} \tag{7}$$

Where q_e is equilibrium adsorption capacity (mg g^{-1}), Ce is the equilibrium BB dye concentration in the liquid phase (mg L^{-1}), q_m is the maximum adsorption capacity of the Langmuir model (mg g^{-1}); K_L is the Langmuir equilibrium constant (L mg^{-1}); K_F is the Freundlich equilibrium constant ((mg g^{-1}) (mg L^{-1})$^{-1/nF}$), $1/nF$ is the heterogeneity factor, q_s is the maximum adsorption capacity of the Sips model (mg g^{-1}), K_S is the Sips equilibrium constant (L mg^{-1}), and ms the exponent of the Sips model.

The parameters of the models were estimated through nonlinear regression using the Quasi-Newton estimation method. Statistica 7.0 software (Statsoft, USA) was used in the calculations. The fit quality was evaluated by determination coefficient (R^2), adjusted determination coefficient (R^2_{adj}), and average relative error (ARE).

References

1. Ho, Y.S.; McKay, G. A Comparison of Chemisorption Kinetic Models Applied to Pollutant Removal on Various Sorbents. *Process Saf. Environ. Prot.* **1998**, *76*, 332–340, doi:10.1205/095758298529696.
2. Langmuir, I. The adsorption of gases on plane surfaces of glass, mica and platinum. *J. Am. Chem. Soc.* **1918**, *40*, 1361–1403, doi:10.1021/ja02242a004.
3. Freundlich, H.M.F. Über die Adsorption in Lösungen. *Zeitschrift Phys. Chemie* **1907**, *57U*, 385–470, https://doi.org/10.1515/zpch-1907-5723.
4. Sips, R. On the Structure of a Catalyst Surface. *J. Chem. Phys.* **1948**, *16*, 490–495, https://doi.org/10.1063/1.1746922.
